# Prevalence of dysmenorrhea among reproductive age group in Saudi Women

**DOI:** 10.1186/s12905-022-01654-9

**Published:** 2022-03-19

**Authors:** Hanadi Bakhsh, Eatedal Algenaimi, Raghad Aldhuwayhi, Maha AboWadaan

**Affiliations:** 1 Clinical Sciences Department, College of Medicine, Princess Nourah Bint Abdulrahman University, Riyadh, Saudi Arabia; 2 Obstetrics and Gynecology Department, King Abdulaziz Medical City, Ministry of National Guards Health Affairs, Riyadh, Saudi Arabia; 3 Obstetrics and Gynecology Department, King Abdullah bin Abdulaziz University Hospital, Princess Nourah Bint Abdulrahman University, Riyadh, Saudi Arabia; 4 Emergency Department, King Faisal Specialist Hospital and Research Center, Riyadh, Saudi Arabia

**Keywords:** Dysmenorrhea, Prevalence, Reproductive Age, Saudi Arabia, Women

## Abstract

**Background:**

The condition of recurrent, crampy, lower abdominal pain during menses is defined as dysmenorrhea. The study aims to assess the factors affecting the prevalence of primary and secondary dysmenorrhea among Saudi women from the reproductive age group.

**Methods:**

A cross-sectional survey-based study recruited 1199 participants through a systematic random sampling technique. The study was carried out among the reproductive age group in Saudi women (total number of 1199) who are more than 18-year-old and less than 45-year-old in Riyadh, King Dom of Saudi Arabia, using an electronic questionnaire.

**Results:**

The observed dysmenorrhea in the study; 1107 (92.3%) women had non-pathological dysmenorrhea (primary) while 92 (7.7%) women had pathological dysmenorrhea (secondary) respectively.

**Conclusion:**

In the present study, the prevalence of dysmenorrhea was high among the recruited Saudi women. The study suggests the inclusion of health education programs for students at the school and university level to deal with problems associated with dysmenorrhea that limit their interference with the student’s life.

## Background

The crampy, recurrent pain in the lower abdomen during menses is defined as dysmenorrhea [1]. Dysmenorrhea is divided into two broad categories, i.e., primary and secondary dysmenorrhea. The presence of crampy, recurrent pain in the lower abdomen during menses in the absence of demonstrable disease is primary dysmenorrhea. Adolescents and young women are more likely to be diagnosed with primary dysmenorrhea, an exclusionary diagnosis. Women suffer from pain related symptoms in secondary dysmenorrhea, with a disorder accounting for symptoms like endometriosis, uterine fibroids, or adenomyosis. The significant clinical features experienced by women suffering from secondary dysmenorrhea include pain during intercourse, resistance to effective treatment, and enlarged uterus [2].

Prostaglandins play a significant role in inducing uterine contractions released from endometrial sloughing at the start of menses [3, 4]. The contractions occur at a frequency of > 4–5 per minute (high frequency) and are incoordinate and nonrhythmic. These contractions result in increased intrauterine pressures, which may even exceed 400 mmHg (ranging between 150 and 180 mmHg) [5]. There is the development of uterine ischemia and accumulation of anaerobic metabolites as uterine pressure exceeds the arterial pressure stimulating type C pain neurons that cause dysmenorrhea. The pain perception can also be determined through stretch receptors’ activation.

Dysmenorrhea is a common problem, and it is experienced by 50–90% of women in their reproductive years worldwide, describing having painful menstruation [6]. Young women with primary dysmenorrhea make the majority group of these women. Primary dysmenorrhea tends to decrease with advancing age [7]. However, secondary dysmenorrhea develops later in life [7]. The dysmenorrhea prevalence among Saudi young women ranges from 60.9 to 89.7% [8–13]. These studies were performed in different cities in Saudi Arabia [8–13]. Dysmenorrhea-associated risk factors include younger age (adolescents in particular), smoking, and stress [14, 15]. Risk reduction is accompanied by hormonal contraceptives, higher parity, and having the first childbirth at a younger age [16, 17]. The severity of dysmenorrhea ranges from mild to severe [1]. Patients with dysmenorrhea often report depressed mood, anger, eating more than usual, nausea, dizziness, headache, fatigue, diarrhoea, or constipation associated with dysmenorrhea [12].

To attain targeted intervention and timely prevalence, it is significant to understand the disease burden among the population. In a similar context, the present study aims to determine factors affecting dysmenorrhea prevalence (primary and secondary) among Saudi women from the reproductive age group. The secondary objectives of the study are as follows;

- To determine the relationship between dysmenorrhea and intake of dairy products.
- To determine the relationship between dysmenorrhea and exercise
- To evaluate the effects of dysmenorrhea on the quality of life

## Methods

### Study design and sampling

A cross-sectional survey-based study was conducted, and the participants were recruited through a systematic random sampling technique. The study was carried out among 1199 Saudi women. Epi Info was used to calculate the sample size at a 95% confidence level.

### Inclusion criteria

The inclusion criteria for the study was Saudi women aged between 18 and 45 years and visiting the private clinic to undergo gynaecological examination for dysmenorrhea problems in Riyadh, King Dom of Saudi Arabia.

### Study tool

The self-administered online questionnaire was used as a study tool on Google Forms. This type of survey was easy to administer and could include many participants.

### Data collection

The study took place between July and September 2020.

The questionnaire was translated into validated Arabic language and back-translated into English. The questionnaire contained many parts, including sociodemographic questions such as (age, marital status, weight, height), menses related information such as (age at menarche, duration of menstrual cycle in last 12 months, regularity of menstrual cycle, duration of the menstrual flow, number of used pads, type of dysmenorrhea) and any medical or psychological illness. Other questions included the severity of menstrual cramps and treatment, possible symptoms associated with dysmenorrhea, general assessment of dairy products intake, and the effects of dysmenorrhea on activities of daily living.

### Data analysis

The data gathered from the survey were entered on a Microsoft excel sheet and then analysed using Statistical Package of Social Sciences (SPSS) version 25 (SPSS Inc., Chicago, IL, USA). Frequencies and percentages were used to represent categorical variables; while, means and standard deviations represented continuous variables. Kolmogorov–Smirnov test was used to determine the normality of the tested data. However, groups on normally distributed variables were compared through parametric tests; while, skewed data was represented through non-parametric tests. The significant association between variables was determined using the chi-square / Fisher’s exact test, considering that cell expected frequency is < 5. The mean significant differences between the patient's age and the dysmenorrhea group were determined using an independent sample t-test. The results were considered statistically significant with a *P* value < 0.05.

## Results

Table 1 shows the demographic characteristics of 1199 women recruited in the study according to inclusion criteria. The mean age of women observed was 27.49 years, with a mean age of 12.76 years menarche. Concerning past medical history and psychological illness; 870 (72.6%) patients had not any past medical history, whereas 32 (2.7%) had irritable bowel syndrome (IBS), 35 (2.9%) had depression, and 81 (6.8%) had more than one medical or psychological illness (Table 2).

**Table 1:** Demographic profile of patients (n = 1199)

Characteristics	Description	N (%)
Marital status	Single	792 (66.1%)
	Married	367 (30.6%)
	Widow	7 (0.6%)
	Divorced	33 (2.8%)
Age (years)	Mean ± SD	27.49 ± 7.63
Age at menarche (years)	Mean ± SD	12.76 ± 1.60
Weight (kg)	Mean ± SD	62.50 ± 17.07
Height (cm)	Mean ± SD	151.14 ± 53.09

**Table 2:** Past medical history and psychological illness of patients

Characteristics	Description	N (%)
I don’t have any medical or psychological illness	Yes	870 (72.6%)
	No	329 (27.4%)
Diabetes Mellitus	Yes	10 (0.8%)
	No	1189 (99.2%)
IBS	Yes	32 (2.7%)
	No	1167 (97.3%)
Schizophrenia	Yes	3 (0.3%)
	No	1196 (99.7%)
Obsessive–compulsive disorder	Yes	1 (0.1%)
	No	1198 (99.9%)
Attention deficit hyperactivity disorder	Yes	5 (0.4%)
	No	1194 (99.6%)
Depression	Yes	35 (2.9%)
	No	1164 (97.1%)
Stress	Yes	27 (2.3%)
	No	1172 (97.7%)
Migraine	Yes	8 (0.7%)
	No	1191 (99.3%)
More than one medical or psychological illness right now	Yes	81 (6.8%)
	No	1118 (93.2%)

Table 3 shows a descriptive analysis of the menstrual cycle, regularity, and type of dysmenorrhea. According to it, 296 (24.7%) women had irregular menstrual cycles, and 103 (8.6%) had less than 21 days of the menstrual cycle, and 76 (6.3%) had irregular bleeding. The majority of them, 979 (81.7%), had the duration of the menstrual flow between 3 and 7 days. Around 92 (7.7%) women had pathological dysmenorrhea.

**Table 3:** Descriptive analysis of menstrual cycle, regularity, and type of dysmenorrhea

Characteristics	Description	N (%)
Regularity of menstrual cycle	Not regular	296 (24.7%)
	Regular	903 (75.3%)
Duration of the menstrual cycle in the last 12 months (number of days from the first day of the menstrual cycle to the first day of the next menstrual cycle)	Less than 21 days between the cycles	103 (8.6%)
	Between 21 and 35 days	907 (75.6%)
	More than 35 days between the cycles	113 (9.4%)
	Irregular bleeding (intermenstrual bleeding) between the cycle	76 (6.3%)
The duration of the menstrual flow	2 days or less	98 (8.2%)
	3–7 days	979 (81.7%)
	8 days or more	122 (10.2%)
Number of used pads	(≤ 2 pads/day) mild	172 (14.3%)
	(3–5 pads/day) moderate	853 (71.1%)
	(≥ 6 pads/day) heavy	174 (14.5%)
Type of dysmenorrhea	Non-pathological cause	1107 (92.3%)
	Pathological cause	92 (7.7%)

Table 4 depicts the analysis of the classification of dysmenorrhea pain, therapy and associated symptoms. Around 170 (14.2%) patients had severe pain. The majority of women, 1086 (88.4%), experienced pain in the lower abdomen, and the majority, 808 (67.4%), had pain for more than 3 days. For relieving pain, only 55 (4.6%) patients used NSAIDs only, and 747 (62.3%) patients used more than one analgesic or other alternative therapies used for relieving menstrual cramps. Considering the possible symptoms associated with dysmenorrhea, abdominal bloating is the most common single symptom that was observed among 35 (2.9%) women with dysmenorrhea, whereas, majority of them, 926 (77.2%), had more than one possible symptom associated with dysmenorrhea (Table 4).

**Table 4:** Descriptive analysis of classification of dysmenorrhea pain, therapy and associated symptoms

Characteristics	Description	N (%)
The intensity of the pain	Mild: (pain that resolves without the need for medication)	402 (33.5%)
	Moderate: (pain that is resolved with simple analgesics (NSAIDs, paracetamol)	571 (47.6%)
	Severe: (pain that is not relieved with simple analgesics and may interfere with usual daily activities)	170 (14.2%)
	None	56 (4.7%)
The pain localisation	Lower abdomen	1060 (88.4%)
	Back	77 (6.4%)
	Thighs	18 (1.5%)
	No pain	44 (3.7%)
The beginning of the pain	Before menstruation	420 (35.0%)
	At the beginning of menstruation	642 (53.5%)
	After the beginning of the menstruation	95 (7.9%)
	No pain	42 (3.5%)
The duration of the pain	1 day	361 (30.1%)
	2 days	27 (2.3%)
	3 days	3 (0.3%)
	> 3 days	808 (67.4%)
The period of pain lasts for	1 day	288 (24.0%)
	2 Days	452 (37.7%)
	3 Days	276 (23.0%)
	More than 3 days	141 (11.8%)
	No pain	42 (3.5%)
Is the pain present after the end of the menstruation	Yes	133 (11.1%)
	No	1066 (88.9%)
Analgesics or alternative therapies used to relieve menstrual cramps (NSAID)	Yes	55 (4.6%)
	No	1144 (95.4%)
Analgesics or alternative therapies used to relieve menstrual cramps (Paracetamol)	Yes	64 (5.3%)
	No	1135 (94.7%)
Analgesics or alternative therapies used to relieve menstrual cramps (Herbal remedies)	Yes	43 (3.6%)
	No	1156 (96.4%)
Analgesics or alternative therapies used to relieve menstrual cramps (Hot water bag or Hot pack)	Yes	30 (2.5%)
	No	1169 (97.5%)
Analgesics or alternative therapies used to relieve menstrual cramps (Take Rest)	Yes	17 (1.4%)
	No	1182 (98.6%)
Analgesics or alternative therapies used to relieve menstrual cramps (Antispasmodics)	Yes	6 (0.5%)
	No	1193 (99.5%)
More than one analgesic or alternative therapies used to relieve menstrual cramps	Yes	747 (62.3%)
	No	452 (37.7%)
No Analgesics or alternative therapies used to relieve menstrual cramps	Yes	237 (19.8%)
	No	962 (80.2%)
Possible symptoms associated with dysmenorrhea (Nausea and Vomiting)	Yes	29 (2.4%)
	No	1170 (97.6%)
Possible symptoms associated with dysmenorrhea (Sweating)	Yes	18 (1.5%)
	No	1181 (98.5%)
Possible symptoms associated with dysmenorrhea (Headache)	Yes	28 (2.3%)
	No	1171 (97.7%)
Possible symptoms associated with dysmenorrhea (Abdominal bloating)	Yes	35 (2.9%)
	No	1164 (97.1%)
Possible symptoms associated with dysmenorrhea (Diarrhea)	Yes	17 (1.4%)
	No	1182 (98.6%)
Possible symptoms associated with dysmenorrhea (Fatigue)	Yes	5 (0.4%)
	No	1194 (99.6%)
Possible symptoms associated with dysmenorrhea (Dizziness)	Yes	4 (0.3%)
	No	1195 (99.7%)
Possible symptoms associated with dysmenorrhea (Mood swing)	Yes	22 (1.8%)
	No	1177 (98.2%)
More than one possible symptom associated with dysmenorrhea	Yes	926 (77.2%)
	No	273 (22.8%)

Table 5 displays the distribution of dysmenorrhea limitation, academic performance, exercise and diet during menstruation. The different stress level was found during menstruation, where the majority, 614 (51.2%) of the women, usually had stress. Regarding limitation during menstruation, 161 (13.4%) women with dysmenorrhea reported to have a physical limitation, and 543 (45.3%) reported to have more than one limitation. Likewise, 122 (10.2%) reported dysmenorrhea affecting their concentration aspect of academic performance, and 462 (38.5%) patients had more than one factor affecting their academic performance. Around more than 512 (42.7%) women reported changes in sleeping routine. Furthermore, 268 (22.4%) performed more than one exercise during one exercise, and 260 (21.7%) reported exercise to reduce period pain. In connection with diet, 745 (62.1%) had all types of diet during the period.

**Table 5:** Distribution of dysmenorrhea limitation, academic performance, exercise and diet during menstruation

Characteristics	Description	N (%)
Stress level during menstruation	Usually	614 (51.2%)
	Often	345 (28.8%)
	Occasionally	180 (15.0%)
	Hardly ever	60 (5.0%)
Any limitations due to the dysmenorrhea (Physical limitation)	Yes	161 (13.4%)
	No	1038 (86.6%)
Any limitations due to the dysmenorrhea (Emotional limitation)	Yes	88 (7.3%)
	No	1111 (92.7%)
Any limitations due to the dysmenorrhea (Social functioning limitation)	Yes	28 (2.3%)
	No	1171 (97.7%)
Any limitations due to the dysmenorrhea (Academic performance limitation)	Yes	38 (3.2%)
	No	1161 (96.8%)
No limitations due to the dysmenorrhea	Yes	341 (28.4%)
	No	858 (71.6%)
More than one limitation due to the dysmenorrhea	Yes	543 (45.3%)
	No	656 (54.7%)
If the dysmenorrhea affects your academic performance, which aspects were affected (Attendance)	Yes	112 (9.3%)
	No	1087 (90.7%)
If the dysmenorrhea affects your academic performance, which aspects were affected (Participation)	Yes	24 (2.0%)
	No	1175 (98.0%)
If the dysmenorrhea affects your academic performance, which aspects were affected (Concentration)	Yes	122 (10.2%)
	No	1077 (89.8%)
If the dysmenorrhea affects your academic performance, which aspects were affected (Studying time)	Yes	16 (1.3%)
	No	1183 (98.7%)
Nothing affects academic performance due to dysmenorrhea	Yes	463 (38.6%)
	No	736 (61.4%)
More than one aspect of dysmenorrhea affects your academic performance	Yes	462 (38.5%)
	No	737 (61.5%)
Any changes in sleep hours during menstruation?	None	354 (29.5%)
	Yes, less than normal	333 (27.8%)
	Yes, more than normal	512 (42.7%)
Are you a smoker?	No	1173 (97.8%)
	Yes, less than one pack a day	23 (1.9%)
	Yes, more than 1 pack	3 (0.3%)
How many times a week you do exercise?	1–2	322 (26.9%)
	≤ 3	297 (24.8%)
	None	580 (48.4%)
Which type of exercise you did it during menstruation (A brisk walk)	Yes	347 (28.9%)
	No	852 (71.1%)
Which type of exercise you did it during menstruation (Jogging)	Yes	1 (0.1%)
	No	1198 (99.9%)
Which type of exercise you did it during menstruation (Dancing)	Yes	11 (0.9%)
	No	1188 (99.1%)
Which type of exercise you did it during menstruation (Biking)	Yes	4 (0.3%)
	No	1195 (99.7%)
Which type of exercise you did it during menstruation (Stretching various parts of the body)	Yes	8 (0.7%)
	No	1191 (99.3%)
Which type of exercise you did it during menstruation (Doing yoga)	Yes	10 (0.8%)
	No	1189 (99.2%)
No exercise during menstruation	Yes	550 (45.9%)
	No	649 (54.1%)
More than type of exercise you did it during menstruation	Yes	268 (22.4%)
	No	931 (77.6%)
Does your regular exercise reduce period pain	Yes	260 (21.7%)
	No	310 (25.9%)
	I don't do any exercise	629 (52.5%)
How many meals you eat per day	2 times per day	734 (61.2%)
	3 times per day	388 (32.4%)
	4 times per day	77 (6.4%)
The major part of your diet during the menstruation is Pasta	Yes	15 (1.3%)
	No	1184 (98.7%)
	I don't do any exercise	0 (0.0%)
The major part of your diet during the menstruation is Meat	Yes	17 (1.4%)
	No	1182 (98.6%)
The major part of your diet during the menstruation is Fruit	Yes	25 (2.1%)
	No	1174 (97.9%)
The major part of your diet during the menstruation is Eggs	Yes	27 (2.3%)
	No	1172 (97.7%)
The major part of your diet during the menstruation is Fish	Yes	7 (0.6%)
	No	1192 (99.4%)
The major part of your diet during the menstruation is Chocolate	Yes	264 (22.0%)
	No	935 (78.0%)
The major part of your diet during the menstruation is Dairy products	Yes	61 (5.1%)
	No	1138 (94.9%)
The major part of your diet during the menstruation is Vegetables	Yes	38 (3.2%)
	No	1161 (96.8%)
More than one diet during menstruation	Yes	745 (62.1%)
	No	454 (37.9%)
How many times eats dairy products per day (milk, yogurt, cheese and labanah)	1 time per day	682 (56.9%)
	2 time per day	284 (23.7%)
	3 time per day	51 (4.3%)
	4 time per day or more	6 (0.5%)
	None	176 (14.7%)
How many times did you experience dysmenorrhea pain after eating dairy products?	Usually	53 (4.4%)
	Often	84 (7.0%)
	Occasionally	183 (15.3%)
	Hardly ever	879 (73.3%)
The amount of caffeine you drink during the menstruation?	1–2 Cup	719 (60.0%)
	3–4 Cup	159 (13.3%)
	More than4 cups	57 (4.8%)
	None	264 (22.0%)
How many times did you experience dysmenorrhea pain after caffeine drinks?	Usually,	40 (3.3%)
	Often	80 (6.7%)
	Occasionally	192 (16.0%)
	Hardly ever	887 (74.0%)

Table 6 shows no statistically significant association among age, marital status, diabetes mellitus, IBS, Schizophrenia, and OCD with primary and secondary dysmenorrhea. However, most 775 (70%) women with age less than 30 years and the majority of the single women, 729 (65.9%), had primary dysmenorrhea. Around 55 (59.8%) women who had irregular menstrual cycles had significantly secondary dysmenorrhea (*P* < 0.001). Similarly, there was a significant association between the menstrual cycle duration in the last 12 months and primary and secondary dysmenorrhea (*P* < 0.001). Here, 11 (12.0%) women with less than 21 days cycle had secondary dysmenorrhea compared to 92 (8.3%) women with less than 21 days cycle with primary dysmenorrhea. Likewise, duration of the menstruation flow was significantly associated with the type of dysmenorrhea, were among women with more than 7 days cycle, 103 (9.3%) had primary and 19 (20.7%) had secondary dysmenorrhea (*P* = 0.002). Moreover, around 801 (72.4%) women with primary and 52 (56.5%) with secondary dysmenorrhea significantly used 3–5 pads per day (*P* = 0.004).

**Table 6:** Impact and Association between dysmenorrhea and clinical and demographic characteristics of patients

Characteristics	Description	Primary dysmenorrhea (n = 1107)	Secondary dysmenorrhea (n = 92)	*P* value
Age	Mean ± SD	27.46 ± 7.65	27.95 ± 7.48	0.548
Age group	< 30	775 (70.0%)	57 (62.0%)	0.228
	30–40	232 (21.0%)	26 (28.3%)	
	> 40	100 (9.0%)	9 (9.8%)	
Marital status	Single	729 (65.9%)	63 (68.5%)	0.577
	Married	342 (30.9%)	25 (27.2%)	
	Widow	7 (0.6%)	0 (0.0%)	
	Divorced	29 (2.6%)	4 (4.3%)	
Diabetes mellitus	Yes	9 (0.8%)	1 (1.1%)	0.781
	No	1098 (99.2%)	91 (98.9%)	
IBS	Yes	29 (2.6%)	3 (3.3%)	0.714
	No	1078 (97.4%)	89 (96.7%)	
Schizophrenia	Yes	2 (0.2%)	1 (1.1%)	0.095
	No	1105 (99.8%)	91 (98.9%)	
Obsessive–compulsive disorder	Yes	1 (0.1%)	0 (0.0%)	0.773
	No	1106 (99.9%)	92 (100.0%)	
Attention deficit hyperactivity disorder	Yes	3 (0.3%)	2 (2.2%)	0.006*
	No	1104 (99.7%)	90 (97.8%)	
Depression	Yes	33 (3.0%)	2 (2.2%)	0.659
	No	1074 (97.0%)	90 (97.8%)	
Stress	Yes	25 (2.3%)	2 (2.2%)	0.958
	No	1082 (97.7%)	90 (97.8%)	
Migraine	Yes	8 (0.7%)	0 (0.0%)	0.413
	No	1099 (99.3%)	92 (100.0%)	
More than one medical or psychological illness right now	Yes	69 (6.2%)	12 (13.0%)	0.012*
	No	1038 (93.8%)	80 (87.0%)	
Regularity of menstrual cycle	Not regular	241 (21.8%)	55 (59.8%)	< 0.001*
	Regular	866 (78.2%)	37 (40.2%)	
Duration of menstrual cycle in the last 12 months (number of days from first day of menstrual cycle to the first day of the next menstrual cycle)	Less than 21 days between the cycle	92 (8.3%)	11 (12.0%)	< 0.001*
	Between 21 and 35 days	865 (78.1%)	42 (45.7%)	
	More than 35 days between the cycle	92 (8.3%)	21 (22.8%)	
	Irregular bleeding (intermenstrual bleeding) between the cycle	58 (5.2%)	18 (19.6%)	
The duration of the menstrual flow	2 days or less	90 (8.1%)	8 (8.7%)	*0.002
	3–7 days	914 (82.6%)	65 (70.7%)	
	8 days or more	103 (9.3%)	19 (20.7%)	
Number of used pads	(≤ 2 pads/day) mild	150 (13.6%)	22 (23.9%)	*0.004
	(3–5 pads/day) moderate	801 (72.4%)	52 (56.5%)	
	(≥ 6 pads/day) heavy	156 (14.1%)	18 (19.6%)	

Table 7 shows a statistically significant association between the intensity of pain and primary and secondary dysmenorrhea (*P* = 0.006). Where majority of 539 (48.7%) women with primary dysmenorrhea had mild and 32 (34.8%) with secondary dysmenorrhea had moderate pain. Moreover, no significant association was demonstrated between the type of dysmenorrhea and pain localisation, duration and period of pain, and use of NSAIDs or paracetamols. Whereas, majority of the women with both types significantly did not use alternative therapies (hot pack) to relieve menstrual cramps (*P* = 0.013). There was no statistically significant association between dysmenorrhea and associated symptoms, limitation and affected academic performance (Table 8).

**Table 7:** Impact and Association between dysmenorrhea and intensity of pain and its management

Characteristics	Description	Primary dysmenorrhea (n = 1107)	Secondary dysmenorrhea (n = 92)	*P*-value
The intensity of the pain	Mild	371 (33.5%)	31 (33.7%)	*0.006
	Moderate	539 (48.7%)	32 (34.8%)	
	Severe	147 (13.3%)	23 (25.0%)	
	No pain	50 (4.5%)	6 (6.5%)	
The pain localisation	Lower abdomen	984 (88.9%)	76 (82.6%)	0.221
	Back	67 (6.1%)	10 (10.9%)	
	Thighs	17 (1.5%)	1 (1.1%)	
	No pain	39 (3.5%)	5 (5.4%)	
The beginning of the pain	Before menstruation	392 (35.4%)	28 (30.4%)	0.602
	At the beginning of menstruation	590 (53.3%)	52 (56.5%)	
	After the beginning of the menstruation	86 (7.8%)	9 (9.8%)	
	No pain	39 (3.5%)	3 (3.3%)	
The duration of the pain	1 day	329 (29.7%)	32 (34.8%)	0.178
	2 days	24 (2.2%)	3 (3.3%)	
	3 days	3 (0.3%)	0 (0.0%)	
	> 3 days	751 (67.8%)	57 (62.0%)	
The period of pain lasts for	1 day	272 (24.6%)	16 (17.4%)	0.943
	2 Days	420 (37.9%)	32 (34.8%)	
	3 Days	252 (22.8%)	24 (26.1%)	
	More than 3 days	124 (11.2%)	17 (18.5%)	
	No pain	39 (3.5%)	3 (3.3%)	
Is the pain present after the end of the menstruation	Yes	123 (11.1%)	10 (10.9%)	0.909
	No	984 (88.9%)	82 (89.1%)	
Analgesics or alternative therapies used to relieve menstrual cramps (NSAID)	Yes	51 (4.6%)	4 (4.3%)	0.66
	No	1056 (95.4%)	88 (95.7%)	
Analgesics or alternative therapies used to relieve menstrual cramps (Paracetamol)	Yes	60 (5.4%)	4 (4.3%)	0.861
	No	1047 (94.6%)	88 (95.7%)	
Analgesics or alternative therapies used to relieve menstrual cramps (Herbal remedies)	Yes	40 (3.6%)	3 (3.3%)	0.11
	No	1067 (96.4%)	89 (96.7%)	
Analgesics or alternative therapies used to relieve menstrual cramps (Hot water bag or Hot pack)	Yes	30 (2.7%)	0 (0.0%)	*0.013
	No	1077 (97.3%)	92 (100.0%)	
Analgesics or alternative therapies used to relieve menstrual cramps (Take Rest)	Yes	13 (1.2%)	4 (4.3%)	0.407
	No	1094 (98.8%)	88 (95.7%)	
Analgesics or alternative therapies used to relieve menstrual cramps (Antispasmodics)	Yes	5 (0.5%)	1 (1.1%)	0.943
	No	1102 (99.5%)	91 (98.9%)	
More than one analgesic or alternative therapies used to relieve menstrual cramps	Yes	690 (62.3%)	57 (62.0%)	0.686
	No	417 (37.7%)	35 (38.0%)

**Table 8:** Impact and Association between dysmenorrhea and associated symptoms, limitation and effected academic performance

Characteristics	Description	Primary dysmenorrhea (n = 1107)	Secondary dysmenorrhea (n = 92)	*P*-value
Possible symptoms associated with dysmenorrhea (Nausea and Vomiting)	Yes	28 (2.5%)	1 (1.1%)	0.387
	No	1079 (97.5%)	91 (98.9%)	
Possible symptoms associated with dysmenorrhea (Sweating)	Yes	16 (1.4%)	2 (2.2%)	0.581
	No	1091 (98.6%)	90 (97.8%)	
Possible symptoms associated with dysmenorrhea (Headache)	Yes	24 (2.2%)	4 (4.3%)	0.183
	No	1083 (97.8%)	88 (95.7%)	
Possible symptoms associated with dysmenorrhea (Abdominal bloating)	Yes	33 (3.0%)	2 (2.2%)	0.659
	No	1074 (97.0%)	90 (97.8%)	
Possible symptoms associated with dysmenorrhea (Diarrhoea)	Yes	16 (1.4%)	1 (1.1%)	0.780
	No	1091 (98.6%)	91 (98.9%)	
Possible symptoms associated with dysmenorrhea (Fatigue)	Yes	4 (0.4%)	1 (1.1%)	0.299
	No	1103 (99.6%)	91 (98.9%)	
Possible symptoms associated with dysmenorrhea (Dizziness)	Yes	4 (0.4%)	0 (0.0%)	0.564
	No	1103 (99.6%)	92 (100.0%)	
Possible symptoms associated with dysmenorrhea (Mood swing)	Yes	21 (1.9%)	1 (1.1%)	0.578
	No	1086 (98.1%)	91 (98.9%)	
More than one possible symptom associated with dysmenorrhea	Yes	856 (77.3%)	70 (76.1%)	0.785
	No	251 (22.7%)	22 (23.9%)	
Stress level during menstruation	Usually	561 (50.7%)	53 (57.6%)	0.438
	Often	325 (29.4%)	20 (21.7%)	
	Occasionally	165 (14.9%)	15 (16.3%)	
	Hardly ever	56 (5.1%)	4 (4.3%)	
Any limitations due to the dysmenorrhea (Physical limitation)	Yes	152 (13.7%)	9 (9.8%)	0.285
	No	955 (86.3%)	83 (90.2%)	
Any limitations due to the dysmenorrhea (Emotional limitation)	Yes	81 (7.3%)	7 (7.6%)	0.917
	No	1026 (92.7%)	85 (92.4%)	
Any limitations due to the dysmenorrhea (Social functioning limitation)	Yes	24 (2.2%)	4 (4.3%)	0.183
	No	1083 (97.8%)	88 (95.7%)	
Any limitations due to the dysmenorrhea (Academic performance limitation)	Yes	37 (3.3%)	1 (1.1%)	0.235
	No	1070 (96.7%)	91 (98.9%)	
No limitations due to the dysmenorrhea	Yes	317 (28.6%)	24 (26.1%)	0.602
	No	790 (71.4%)	68 (73.9%)	
More than one limitation due to the dysmenorrhea	Yes	496 (44.8%)	47 (51.1%)	0.244
	No	611 (55.2%)	45 (48.9%)	
If the dysmenorrhea affects your academic performance, which aspects were affected (Attendance)	Yes	102 (9.2%)	10 (10.9%)	0.600
	No	1005 (90.8%)	82 (89.1%)	
If the dysmenorrhea affects your academic performance, which aspects were affected (Participation)	Yes	23 (2.1%)	1 (1.1%)	0.514
	No	1084 (97.9%)	91 (98.9%)	
If the dysmenorrhea affects your academic performance, which aspects were affected (Concentration)	Yes	112 (10.1%)	10 (10.9%)	0.818
	No	995 (89.9%)	82 (89.1%)	
If the dysmenorrhea affects your academic performance, which aspects were affected (Studying time)	Yes	16 (1.4%)	0 (0.0%)	0.246
	No	1091 (98.6%)	92 (100.0%)	
Nothing affects academic performance due to dysmenorrhea	Yes	427 (38.6%)	36 (39.1%)	0.915
	No	680 (61.4%)	56 (60.9%)	
More than one aspect of dysmenorrhea affects your academic performance	Yes	427 (38.6%)	35 (38.0%)	0.920
	No	680 (61.4%)	57 (62.0%)	
Any changes in sleep hours during menstruation?	None	329 (29.7%)	25 (27.2%)	0.865
	Yes, less than normal	306 (27.6%)	27 (29.3%)	
	Yes, more than normal	472 (42.6%)	40 (43.5%)	
Are you a smoker?	No	1086 (98.1%)	87 (94.6%)	0.051
	Yes, less than one pack a day	19 (1.7%)	4 (4.3%)	
	Yes more than 1 pack	2 (0.2%)	1 (1.1%)	
Number of cigarettes	Mean ± SD	2.87 ± 1.18	6.67 ± 2.50	0.628

There was a statistically significant association between dysmenorrhea type and dietary habits, exercise and quality of life, as shown in Table 9. There was a statistically significant association between exercise during menstruation and primary and secondary dysmenorrhea (*P* = 0.001). It is shown that 1105 (99.8%) patients from primary and 90 (97.8%) secondary dysmenorrhea did not use bikes, respectively.

**Table 9:** Impact and association between dysmenorrhea and dietary habits, exercise and quality of life

Characteristics	Description	Primary dysmenorrhea (n = 1107)	Secondary dysmenorrhea (n = 92)	*P*-value
How many times a week you do exercise?	1–2	299 (27.0%)	23 (25.0%)	0.832
	≤ 3	272 (24.6%)	25 (27.2%)	
	None	536 (48.4%)	44 (47.8%)	
Which type of exercise you did it during menstruation (A brisk walk)	Yes	323 (29.2%)	24 (26.1%)	0.529
	No	784 (70.8%)	68 (73.9%)	
Which type of exercise you did it during menstruation (Jogging)	Yes	1 (0.1%)	0 (0.0%)	0.773
	No	1106 (99.9%)	92 (100.0%)	
Which type of exercise you did it during menstruation (Dancing)	Yes	11 (1.0%)	0 (0.0%)	0.337
	No	1096 (99.0%)	92 (100.0%)	
Which type of exercise you did it during menstruation (Biking)	Yes	2 (0.2%)	2 (2.2%)	*0.001
	No	1105 (99.8%)	90 (97.8%)	
Which type of exercise you did it during menstruation (Stretching various parts of the body)	Yes	7 (0.6%)	1 (1.1%)	0.607
	No	1100 (99.4%)	91 (98.9%)	
Which type of exercise you did it during menstruation (Doing yoga)	Yes	8 (0.7%)	2 (2.2%)	0.141
	No	1099 (99.3%)	90 (97.8%)	
No exercise during menstruation	Yes	503 (45.4%)	47 (51.1%)	0.296
	No	604 (54.6%)	45 (48.9%)	
More than type of exercise you did it during menstruation	Yes	252 (22.8%)	16 (17.4%)	0.234
	No	855 (77.2%)	76 (82.6%)	
Does your regular exercise reduce period pain	Yes	243 (22.0%)	17 (18.5%)	0.701
	No	284 (25.7%)	26 (28.3%)	
	I don't do any exercise	580 (52.4%)	49 (53.3%)	
How many meals you eat per day	2 times per day	681 (61.5%)	53 (57.6%)	0.078
	3 times per day	360 (32.5%)	28 (30.4%)	
	4 times per day	66 (6.0%)	11 (12.0%)	
The major part of your diet during the menstruation is Pasta	Yes	15 (1.4%)	0 (0.0%)	0.261
	No	1092 (98.6%)	92 (100.0%)	
The major part of your diet during the menstruation is Meat	Yes	16 (1.4%)	1 (1.1%)	0.78
	No	1091 (98.6%)	91 (98.9%)	
The major part of your diet during the menstruation is Fruit	Yes	24 (2.2%)	1 (1.1%)	0.486
	No	1083 (97.8%)	91 (98.9%)	
The major part of your diet during the menstruation is Eggs	Yes	27 (2.4%)	0 (0.0%)	0.13
	No	1080 (97.6%)	92 (100.0%)	
The major part of your diet during the menstruation is Fish	Yes	7 (0.6%)	0 (0.0%)	0.444
	No	1100 (99.4%)	92 (100.0%)	
The major part of your diet during the menstruation is Chocolate	Yes	240 (21.7%)	24 (26.1%)	0.327
	No	867 (78.3%)	68 (73.9%)	
The major part of your diet during the menstruation is Dairy products	Yes	58 (5.2%)	3 (3.3%)	0.407
	No	1049 (94.8%)	89 (96.7%)	
The major part of your diet during the menstruation is Vegetables	Yes	35 (3.2%)	3 (3.3%)	0.958
	No	1072 (96.8%)	89 (96.7%)	
More than one diet during menstruation	Yes	685 (61.9%)	60 (65.2%)	0.525
	No	422 (38.1%)	32 (34.8%)	
How many time eats dairy products per day (milk, yogurt, cheese and labanah)	1 time per day	629 (56.8%)	53 (57.6%)	0.698
	2 time per day	266 (24.0%)	18 (19.6%)	
	3 time per day	47 (4.2%)	4 (4.3%)	
	4 time per day or more	6 (0.5%)	0 (0.0%)	
	None	159 (14.4%)	17 (18.5%)	
How many times did you experience dysmenorrhea pain after eating dairy products?	Usually	47 (4.2%)	6 (6.5%)	0.754
	Often	78 (7.0%)	6 (6.5%)	
	Occasionally	168 (15.2%)	15 (16.3%)	
	Hardly ever	814 (73.5%)	65 (70.7%)	
The amount of caffeine you drink during the menstruation?	1–2 Cup	669 (60.4%)	50 (54.3%)	0.726
	3–4 Cup	145 (13.1%)	14 (15.2%)	
	More than4 cups	52 (4.7%)	5 (5.4%)	
	None	241 (21.8%)	23 (25.0%)	
How many times did you experience dysmenorrhea pain after caffeine drinks?	Usually	37 (3.3%)	3 (3.3%)	0.863
	Often	73 (6.6%)	7 (7.6%)	
	Occasionally	180 (16.3%)	12 (13.0%)	
	Hardly ever	817 (73.8%)	70 (76.1%)	

## Discussion

The study aims to determine the factors affecting dysmenorrhea (primary and secondary) prevalence among Saudi women from the reproductive age group. Dysmenorrhea is an important symptom among many women of reproductive age. Dysmenorrhea has a significant impact on the health-related quality of life, work productivity, and health-care utilisation. The dysmenorrhea prevalence came out to be 95.3% in the present study. This prevalence was higher than that observed in the other studies in Saudi Arabia and worldwide [8–13]. The worldwide prevalence ranged from 50 to 90% [18–21]. The high majority observed in this study could be due to the different population age groups included in it or that the women who had the symptoms were more interested in taking part in it.

The study also determined the relationship between dysmenorrhea and intake of dairy products, which showed no significant association. A systematic review conducted by Zahra et al. [22] found that fruits, vegetables, milk, fish and dairy products had positive associations with decreased menstrual pain in primary dysmenorrhea. However, the majority of the participants did not have fruits, vegetables, and dairy products as part of their primary diet, which could be why there was a high prevalence of dysmenorrhea in the studied population.

Also, this study had the objective to elucidate the possible relationship between exercise and dysmenorrhea and to evaluate the impact of dysmenorrhea on the quality of life. Varied researches previously have shown dysmenorrhea to be impacted by multiple factors, of these were physical and daily activities, emotional health, social activities, family and friends’ relationships, in addition to the academic performance regarding concentration, attendance, class participation, and study time [23, 24]. In this study, the impact and association between dysmenorrhea and associated symptoms, limitation and affected academic performance were not statistically significant. As most of the women included in the study reported not to have done an adequate amount of exercise, this could be another factor that the prevalence among participants was high.

Another notable finding of this study was the significant association between dysmenorrhea and irregularity of the menstrual cycle. Similarly, a survey by Ameade et al. [25] showed a statistically significant association between the severity of dysmenorrhea and irregularity of menstruation. Also, the menstrual flow and dysmenorrhea were necessary, similar to the results of previous studies [26, 27]. There was a statistically significant association between the type of dysmenorrhea and pain intensity (*P* = 0.006). The severity of pain was high among women with secondary dysmenorrhea compared to women with primary dysmenorrhea.

Menstrual abnormalities, dysmenorrhea, infertility, chronic pelvic pain (CPP), and dyspareunia are endometriosis's most prevalent clinical indications. Endometriosis symptoms frequently impact patients' social and psychological functioning. As a result, endometriosis is considered a debilitating disease that can jeopardise social interactions and mental health [28]. Endometriosis can be effectively treated with progestins. The effects of the etonogestrel implant on pelvic discomfort, sexual function, and quality of life in women needing long-term reversible contraception and having ovarian cysts of possible endometriotic origin are investigated in the study by Sansone et al. [29]. In patients with ovarian cysts suspected of being caused by endometriosis, etonogestrel implants appear to relieve pelvic discomfort, enhance sexual function, and improve quality of life. Endometriosis is characterised by endometrial-like tissue outside the uterus, which is accompanied by a persistent and inflammatory response. Brasil et al. [30] determined the prevalence and degrees of psychological stress among endometriosis patients. The study showed that multidisciplinary illness management should include mental health assistance inpatient care beyond pain treatment. Moreover, the medical team’s attitude toward the patients’ psychological stress may positively impact their therapy.

It is essential to encourage modifications in the diet and lifestyle of individuals like restricted intake of salt and excessive caffeinated drinks with effective exercising for reducing the severity of dysmenorrhea symptoms. The possible side effects of using analgesics also need to be informed to the women, and they need to be encouraged for other management techniques like the use of hot pads [31, 32]. Measures to deal with dysmenorrhea need to be focused at the school and university level for limiting its interference with the student’s life. Apart from these implications, there were some limitations of this study. For instance, the data was collected using self-administered questionnaires (electronic questionnaires), which decreased the reliability of the results. Moreover, the study only included females from a specific region. The study also fails to compare the sample to the number of Saudi women of reproductive age. As a result, the results just reflect a small portion of the sample. Future studies need to include females from other regions of Saudi Arabia to generalise the results to all Saudi females.

## Conclusions

The present study revealed an increased prevalence of dysmenorrhea among Saudi women of reproductive age. This could be due to an unbalanced diet and a low level of exercise seen in the studied group. The intensity of pain was high among women with secondary dysmenorrhea compared to women with primary dysmenorrhea. There was no association of prevalence of dysmenorrhea with the age group or marital status. Campaigns on the information regarding dysmenorrhea and its remedies should be promoted to make the quality of life of women better that could get limited due to menstruation.

## Data Availability

The datasets used and analysed during the current study are available from the corresponding author on reasonable request.

## Abbreviations

SD: Standard deviation
PNU: Princess Nourah Bint Abdulrahman
